# Exposure to environmental tobacco smoke in non - smoking adults in Israel: results of the second Israel biomonitoring survey

**DOI:** 10.1186/s13584-018-0229-9

**Published:** 2018-06-25

**Authors:** Tamar Berman, Zohar Barnett-Itzhaki, Nisim Mery, Lital Keinan-Boker, Tal Shimony, Rebecca Goldsmith, Thomas Göen, Haim Geva, Laura Rosen

**Affiliations:** 1 Public Health Services, Ministry of Health, 39 Yirmiyahu Street, 9446724 Jerusalem, Israel; 2 Department of Health Promotion, School of Public Health, Sackler Faculty of Medicine, Tel Aviv University, Tel-Aviv, Israel; 3 Bioinformatics Department, School of Life and Health Sciences, Jerusalem College of Technology, Jerusalem, Israel; 4 Israel Center for Disease Control, Israel Ministry of Health, Gertner Institute, Sheba Medical Center, Tel Hashomer, 52621 Ramat Gan, Israel; 5 Institute and Outpatient Clinic for Occupational, Social and Environmental Medicine of the University Erlangen-Nuremberg, Erlangen, Germany

**Keywords:** Environmental tobacco smoke, Urinary cotinine, Human biomonitoring, Exposure

## Abstract

**Background:**

Exposure to environmental tobacco smoke (ETS) increases the risk of heart and respiratory disease, cancer, and premature mortality in non-smoking individuals. Results from the first Israel Biomonitoring Study in 2011 showed that over 60% of non-smoking adults are exposed to ETS. The purpose of the current study was to assess whether policies to restrict smoking in public places have been associated with reductions in exposure to ETS, and to examine predictors of exposure.

**Methods:**

We analyzed urinary cotinine and creatinine concentrations in 194 adult participants in the National Health and Nutrition (RAV MABAT) Survey in 2015–2016. Study participants were interviewed in person on smoking status and exposure to ETS. We calculated creatinine-adjusted and unadjusted urinary cotinine geometric means and medians among smokers and non-smokers. We analyzed associations in univariable analyses, between socio-demographic variables and self – reported exposure, and urinary cotinine concentrations.

**Results:**

There was no reduction in geometric mean urinary cotinine levels in non-smokers in the current study (1.7 μg/g) compared to that in 2011 (1.6 μg/g). Median cotinine levels among the non – smoking Arab participants were higher in comparison to the Jewish and other participants (2.97 versus 1.56 μg/l, *p* = 0.035). Participants who reported that they were exposed to ETS at home had significantly higher median levels of creatinine adjusted urinary cotinine than those reporting they were not exposed at home (4.19 μg/g versus 2.9 μg/g, *p* = 0.0039).

**Conclusions:**

Despite additional restrictions on smoking in public places in 2012–2016, over 60% of non-smoking adults in Israel continue to be exposed to ETS. Urinary cotinine levels in non-smokers have not decreased compared to 2011. Results indicate higher exposure to ETS in Arab study participants and those reporting ETS exposure at home. There is an urgent need: (1) to increase enforcement on the ban on smoking in work and public places; (2) for public health educational programs and campaigns about the adverse health effects of ETS; and (3) to develop and disseminate effective interventions to promote smoke free homes. Periodic surveys using objective measures of ETS exposure (cotinine) are an important tool for monitoring progress, or lack thereof, of policies to reduce exposure to tobacco smoke in non-smokers.

## Background

Environmental tobacco smoke (ETS) is smoke emitted from a burning tobacco product and the smoke exhaled by a smoker. Exposure to ETS has immediate adverse effects on the cardiovascular system and causes premature mortality in non-smoking individuals, as well as a range of heart diseases and respiratory diseases. There is sufficient evidence that ETS causes lung cancer and suggestive evidence that ETS may increase the risk of breast cancer, nasal sinus cavity cancer, and nasopharyngeal cancer in adults [1]. Globally, 603,000 deaths were attributable to ETS exposure in 2004 [2]. In Israel, there were an estimated 790 deaths and estimated 36,049 hospital days attributable to ETS in 2014 [3].

Human biomonitoring (HBM) is the measurement of contaminants or their metabolites in biological samples. HBM is an important tool for assessing exposure to ETS in non-smokers and for measuring the effectiveness of tobacco control strategies aimed at reducing exposure [4]. Cotinine, the primary nicotine metabolite, is frequently used as an ETS biomarker. In contrast to nicotine, which has a biological half-life of 1–3 hours (h), cotinine has a longer biological half- life (16–18 h) and levels remain fairly constant during the day. Cotinine can be measured in serum, urine, saliva, and hair, with urinary cotinine reflecting very recent exposure to ETS occurring in the past few days [5].

An HBM survey in Israel conducted in 2011 revealed that over 60% of adult non-smokers have quantifiable levels of urinary cotinine, indicating widespread exposure to ETS [6]. In 2011, the National Plan for the Reduction of Tobacco Use and Damage was passed by the government; this included extensions of the existing laws on smoke-free places, which passed a Knesset vote in May 2012 and went into effect in July 2012. The ban was extended to entrances to medical facilities, train stations, outdoor swimming pools, and government offices (in 2012). This was extended to sports stadiums in 2014 and schools in 2016 [7]. However, implementation and enforcement in Israel of the ban on smoking in public places is problematic. The aim of this study was to examine whether extension of the ban has been associated with reduced ETS exposure in non-smoking adults in Israel. An additional aim of the study was to assess predictors of ETS exposure in adult non-smokers in Israel.

## Methods

The study included 194 adults (ages 18–64) who participated in the 2015–2016 National Health and Nutrition Survey (RAV MABAT), conducted by the Israel Center for Disease Control (ICDC) and the Nutrition Division at the Israel Ministry of Health, in collaboration with the Central Bureau of Statistics. The aim of the RAV MABAT survey is to collect data on nutritional habits, anthropometric measurements, and health-related behaviors, such as smoking, alcohol consumption and physical activity, in the general Israeli population. The eligible population for the RAV MABAT survey included Israeli adults, ages 18 and over, aiming to represent the Israeli non-institutionalized adult population. The participants in the current study were adult participants in the RAV MABAT study who provided urine samples.

The study was conducted in accordance with the ethical principles of the Declaration of Helsinki. The study protocol was reviewed and approved by the Sheba Tel Hashomer Helsinki Committee. Written informed consent was obtained for all respondents. Participation in the study was voluntary.

Study participants were interviewed in person using a structured questionnaire. The interviews were administered by trained interviewers. The interview consisted of a health and lifestyle questionnaire, demographic questionnaire, a 24 h recall and smoking questionnaire (active smoking including hookah (nargila), smoking in the past, and self- reported exposure to ETS). Participants were asked to estimate their exposure to ETS in the past month (very high/ high/ little/ none) and were asked where they are exposed to ETS (home/ work/ other). The smoking questionnaire has been validated and used previously by the Central Bureau of Statistics.

Smoking status was based on self-report. The question used for active tobacco smoking status was: “Do you currently smoke, including hookah?”. Based on the answer to the question, participants were classified as smokers or non-smokers. Non-smokers included former smokers and participants reporting never smoking.

Urine spot samples were collected in 120-ml urine specimen containers. All urine samples were maintained at below 4 °C for a maximum of 12 h until they were transported to the Asaf Harofeh Medical Center. Urine samples were aliquoted and frozen at −20 °C. Frozen urine samples were transferred to Sheba Medical Center at Tel Hashomer and then were shipped to the University of Erlangen–Nuremberg in Germany on dry ice (−70 °C), where they were analyzed. Researchers at the University of Erlangen–Nuremberg had no access to details on participant’s identification.

Laboratory analyses of cotinine and creatinine were performed at the Institute and Outpatient Clinic of Occupational, Social and Environmental Medicine, University Erlangen-Nuremberg in Germany. Cotinine in urine was determined using a gas chromatography mass spectrometry procedure validated and published by the working group “Analyses in biological materials” [8]. In brief, cotinine was extracted from the urine using dichloromethane and quantified after gas chromatographic separation by mass spectrometry in single ion monitoring mode [9]. Deuterated cotinine was used as an internal standard. Limit of detection (LOD) was 0.5 μg/liter and limit of quantification (LOQ) was 1 μg/liter. Creatinine in urine was determined by photometric detection as picrate according to the Jaffé method [10]. Quality control was performed by analyzing aliquots of control material in each series and accuracy was validated by the successful participation in G-EQUAS for both parameters.

Urinary analyte concentrations were provided in units of μg/liter. In order to correct for variable dilutions among spot samples, these concentrations were divided by urinary creatinine concentrations (g creatinine/l urine) to generate creatinine-adjusted analyte concentrations.

### Statistical methods

Concentrations below the LOQ for cotinine were replaced by the LOD. We calculated percent of non–smokers with urinary cotinine above the LOQ, and geometric mean and median of cotinine in smokers and non-smokers. We conducted all calculations using both unadjusted (μg/liter) and creatinine adjusted (μg/g) values.

Since data were not normally distributed we used non-parametric statistical tests. We used the Mann-Whitney test to compare median creatinine corrected urinary cotinine values in the current study with that from 2011. Cotinine levels in smokers, former smokers, and non-smokers were compared using the Kruskal-Wallis test.

We calculated the proportion of men and women who reported that they were non-smokers in both Arab and Jewish populations. We compared cotinine levels in Arab versus Jewish non-smokers; and male versus female non-smokers using the Mann-Whitney test. In addition, urinary cotinine concentrations were calculated for urinary creatinine-adjusted cotinine concentrations and compared in sub-groups of non-smokers using the t-test procedure for lognormally distributed data.

We compared the proportion of participants reporting ETS exposure at home versus at work or other location, by ethnicity and gender, using the Chi Square test. Finally, we compared urinary cotinine concentrations in non-smokers, and the proportion of participants with urinary cotinine above the LOQ, based on self- reported exposure to ETS, using the Chi Square test. We conducted linear regression analysis using lognormally distributed values. Finally, we calculated Odds Ratios of having urinary cotinine above LOQ in different subgroups in the study (by ethnicity, gender, former smokers, self – reported exposure to smoking).

## Results

Of 194 participants, three were excluded due to unverified smoking status (no answer to question on current smoking in the questionnaire) and two were excluded due to absence of cotinine measurements. The remaining participants were divided into two groups according to their self-report: smokers (*n* = 51) and non-smokers (*n* = 138). In the main analysis, five non-smokers were excluded due to discrepancy between self-reported smoking status as non-smokers and their measured creatinine-adjusted levels of cotinine of over 150 μg/g [11] resulting in 133 non-smokers. The non-smokers were divided to “former smokers” (*n* = 18) and to non-smokers who reported they never smoked (“never smoked”, *n* = 115).

Non-smokers included a higher proportion of women (55% vs. 45%), and the proportion of non-smokers among the Jewish participants was higher than the proportion of non-smokers among the Arab participants (74.3% vs. 62.9%). Table 1 shows the percent of smokers and non-smokers by ethnicity and gender.

**Table 1:** Smoking Rates in Arab, Jewish, and Other Participants, by gender

		Total	Smoking (%)	Non-smoking (%)
Jewish	Males	64	15 (23.4%)	49 (76.5%)
	Females	76	21 (27.6%)	55 (72.4)
Arab	Males	19	10 (52.6%)	9 (47.4%)
	Females	16	3 (18.8%)	13 (81.2%)
Other	Males	3	1	2
	Females	6	1	5
Total	Males	86	26 (30.3%)	60 (69.7%)
	Females	98	25 (25.5%)	73 (74.5%)

The cotinine levels among smokers (1091.74 μg/g) were statistically significantly higher than the levels among non-smokers (3.66 μg/g), *p* < 0.0001. Among smokers, 100% had urinary cotinine above the LOQ, compared to 63.2% of the non-smokers.

The geometric mean (GM) creatinine adjusted cotinine level was 1.7 μg/g, comparable to that in 2011 (Table 2). Median cotinine levels in the current study were significantly higher than in the previous survey in 2011 (*p* = 0.029).

**Table 2:** Percentage of Non-smokers with Quantifiable Urinary Cotinine, Geometric Mean, and Median Urinary Cotinine in Non-smokers, in 2011 and in 2015–2016

Year	2011	2015–2016
Percent of non-smokers with cotinine ≥ LOQ	62.2%	63.2%
Geometric Mean of creatinine adjusted cotinine levels among non-smokers (μg/g)	1.6	1.7
Median creatinine adjusted cotinine levels among non-smokers (μg/g)	1.1	1.8

Cotinine levels were higher among former smokers (adjusted cotinine 4.67 μg/g) in comparison to non-smokers who reported they never smoked (adjusted cotinine 3.5 μg/g, differences not statistically significant).

Cotinine levels among the non–smoking Arab participants were higher in comparison to the Jewish and other participants (Table 3). There was a statistically significant difference between the two group’s (non-adjusted) cotinine levels (*p* = 0.035, Mann-Whitney test). Cotinine levels among non-smoking men were similar to those among non-smoker women.

**Table 3:** Urinary Cotinine Concentrations among Non-smokers by Ethnicity and Gender

Population	Mean cotinine levels (μg/l)	Median cotinine levels (μg/l)	Mean creatinine adjusted cotinine levels (μg/g)	Median creatinine adjusted cotinine levels (μg/g)
Arabs (*n* = 22)	4.94	2.97	3.74	2.06
Jews and others (*n* = 111)	2.93	1.56	3.64	1.71
Men (*n* = 60)	3.37	1.87	3.19	1.63
Women (*n* = 73)	3.18	1.54	4.03	1.95
Overall (*n* = 133)	3.27	1.66	3.66	1.83

71.2% (*n* = 136) of all of the participants (including smokers) reported they were exposed to ETS in the last month. 65.4% of non-smokers (87 of 133) reported they were exposed to ETS in the last month . Of those who reported that they were very exposed to ETS (*N* = 15), 60% reported that they were exposed at home. Participants who reported that they were exposed at home (*N* = 23) had significantly higher levels of urinary cotinine that those reporting they were not exposed at home (4.19 μg/g versus 2.9 μg/g, *p* = 0.0039).

The percent of Arab non-smokers reporting they were exposed to ETS at home (52.9%) was higher than those of the Jewish and other non-smokers (19.7%) (*p* = 0.0113, Chi Square test) (Table 4). The percent of Jewish and other non-smokers reporting they were exposed to ETS at work was higher than those of the Arab non-smokers (46.5% compared to 23.5%, difference not statistically significant). 76.1% of the non-smokers reported they were exposed to ETS in another location (not home or work). The percent of Jewish and other non-smokers reporting they were exposed to ETS at another location was higher than those of the Arab non-smokers (difference not statistically significant).

**Table 4:** Percent of non-smokers exposed to ETS (according to self-report), by location of exposure and ethnicity

Location of exposure to ETS	All non-smokers % (n)	Arab non-smokers % (n)	Jewish and other non-smokers % (n)
Home	26.1 (23)	52.9 (9)	19.7 (14)
Work	42.1 (37)	23.5 (4)	46.5 (33)
Another location	76.1 (67)	70.6 (12)	77.5 (55)

Cotinine levels were detected in 67% of the non-smokers reporting very high exposure to ETS, in comparison to 61.1% who reported high exposure, 63.2% who reported low exposure and 64.4% who reported that they were not exposed to ETS in the last month. Cotinine levels among non-smokers reporting very high exposure to ETS (average cotinine levels of 3.72 μg/l) were not significantly higher than those measured in non-smokers reporting they were not exposed to ETS (average cotinine levels of 2.63 μg/l).

In a univariable analysis, ethnicity, gender, location of exposure to ETS, and self-reported exposure to ETS were not significant predictors of creatinine adjusted urinary cotinine concentrations. When using lognormally distributed values, self- reported exposure at home was a significant predictor of creatinine adjusted urinary cotinine (*p* = 0.03). Odds ratio for having urinary cotinine concentrations above the LOQ were not significantly higher in non-smokers by ethnicity, gender, and extent of self – reported exposure to ETS. Odds ratio for having urinary cotinine concentrations above LOQ was elevated (OR = 4.8) in participants who reported exposure to ETS at home compared to other participants.

As a sensitivity analysis, we included 5 participants who reported that they didn’t smoke, but whose creatinine adjusted urinary cotinine levels were above 150 μg/g, in the group of non-smokers. Three of 5 of these participants reported living with an active smoker. Findings were generally similar to those when excluding these 5 participants. When these five participants were included in the analysis, cotinine urinary concentrations in those reporting very high exposure to ETS was significantly higher (*p* = 0.0378) using t-test procedure for lognormally distributed values.

## Discussion

Our findings indicate that over 60% of the non-smoking adult population in Israel is exposed to ETS. Since our previous survey in 2011, there has been no decrease in exposure to ETS in non-smoking adults. Median adjusted urinary concentrations were in fact significantly higher in 2015–2016 compared to 2011. Results likely reflect exposure to ETS at home and workplaces of the study participants; the fact that there had been no decrease in smoking rates in Israel during this period; and the absence of adequate enforcement by local authorities on restrictions on smoking in public places [12].

There is considerable variability in biomarkers used to measure population-wide ETS exposure (serum cotinine, urinary cotinine, saliva cotinine), which limits the comparability with international studies. Based on data on urinary cotinine in non-smoking adults in the Canadian Health Measures Survey Cycle 4 (2014–2015), 12.7% of non-smokers aged 20–39 years and 9.3% of non-smokers aged 40–59 years had urinary cotinine levels above the level of detection of 1.1 μg/L [13]. In our study, 68.7% of participants ages 20–39 had cotinine levels above 1.1 μg/L, and 56.5% of participants ages 40–59 had cotinine levels above 1.1 μg/L.

Urinary cotinine (unadjusted for creatinine) was higher in Arab participants and a higher proportion of Arab non-smokers reported exposure to ETS at home compared to Jewish and other participants. These findings are consistent with results of the Israeli National Health Interview Survey (INHIS-3) in 2013–2015 in which Arab participants reported higher exposure to ETS compared to Jewish participants (54.7% of Arab women compared to 26.6% of Jewish women; 63.5% of Arab men compared to 30.3% of Jewish men) [14].

There was an indication that ETS exposure was higher in former smokers (although the difference was not significant). This may be related to occasional smoking or increased social interactions with smokers among former smokers. Increased exposure to ETS in former smokers has been reported previously in studies on ETS exposure in Swiss and Korean adults [15, 16].

In the main analysis, urinary cotinine concentrations, and percent of participants with urinary cotinine above the LOQ, were not significantly higher in individuals reporting very high exposure to ETS. In addition, urinary cotinine was quantifiable in 64.4% of the non-smokers who reported they were not exposed to ETS in the past month. There are several possible explanations for these findings. First, participants were asked to evaluate ETS exposure in the past month, whereas urinary cotinine measures reflect very recent exposure (in the days before urine collection). Next, individuals may be unaware of actual exposure [17]. Because 85% of smoke is invisible and smell is an unreliable indicator of exposure, many people may mistakenly believe that they are unexposed, even though they actually are [18, 19].

Previous studies have found poor agreement between self-reported ETS exposure and measurable cotinine, suggesting that self-reported exposure to ETS appears to be unreliable for the purposes of policy evaluation and that objective measures such as cotinine measurement should be used to identify and track the burden of ETS exposure in the population [20]. In the INHIS 2013–2015 survey, 37% of non-smokers reported exposure to ETS at least once or twice a week. Our results indicate that this number does not accurately reflect the extent of ETS exposure in the non-smoking population, and that exposure may be more widespread. We note that direct comparison between self- reported exposure to ETS in the current study and in INHIS surveys is not possible because of differences in the questionnaires and study methodology (phone versus in person interviews).

In contrast to findings from the current study, national human HBM studies on ETS exposure using cotinine measurements have been used by other countries to demonstrate the positive effects of smoke-free legislation and identify its impact on different population groups. For example, in England, levels of ETS exposure among non-smoking adults declined significantly after smokefree legislation was implemented and apparently enforced. After adjusting for prelegislative trends and other factors that may influence exposure, the odds of having cotinine below the level of detection were 1.5 times higher after the legislation and geometric mean cotinine levels fell by 27% [21]. In the US, following increased restrictions on smoking at work and in other public places, and further efforts to reduce the exposure of non-smokers in the home, non-smoker serum cotinine concentrations declined by 70% during 1988–2002 [22]. In Korea, urinary cotinine in non-smokers decreased from 2.61 μg/L in 2009–2011 to 1.38 μg/L in 2012–2014, following the decrease in smoking rate and policies in 2010–2012 to expand restrictions on smoking in public areas [23].

There are several limitations to the current study. First, the HBM study was not a random sample and included only adult participants in the RAV MABAT study who provided a urine sample. The Arab population and men were slightly under-represented in the HBM study. The comparability of results from the current study to those in 2011 is limited by differences in recruitment methods and the study sample. Our relatively small sample size gives limited power to identify differences in magnitude of exposure between specific sub-groups such as those based on ethnicity. In addition, smoking status was based on self-report. We excluded participants who reported they don’t smoke but had urinary cotinine levels higher than 150 μg/g in the main analysis, but included them in the sensitivity analysis. Of note, three of five of these participants reported living with smokers, so it is possible that their urinary cotinine levels reflect high levels of exposure to ETS and not active smoking. However, since once of the aims of this study was to compare findings to those in the 2011 survey on urinary cotinine in non-smokers in Israel, and in that analysis self – reported non-smokers with urinary cotinine above 150 μg/g were excluded, we excluded these 5 participants in the main analysis in the current study as well. Another limitation is the possibility that cotinine levels found in our non-smoking population derived not only from ETS, but, in some cases, from use of nicotine replacement therapy or e-cigarettes. Dietary intake of nicotine from food like fruits and vegetables is possible but likely to be negligible [6, 24]. In addition, the cotinine measurements were based on spot urine samples that represent short-term exposure to ETS. Previous research has indicated that epidemiologic research on ETS exposure can benefit from multiple urine samples [25]. Finally, urinary creatinine concentrations differ dramatically among different demographic groups [26]; thus we compared both adjusted and unadjusted urinary cotinine concentrations. Of note, differences between Arab and other participants were significant only when using values not adjusted for creatinine.

The study findings highlight the need for improved policies to protect non-smokers from exposure to ETS, including expansion of the current restrictions on smoking in public places to outdoor public areas. Indeed, the Ministry of Health plans to extend restrictions on smoking to additional public places including playgrounds and outdoor sports facilities. In addition, there is a need for increased enforcement on the ban on smoking in work and public places. This need is urgent considering indications of a decrease in recent years in enforcement by local authorities of restrictions on smoking in public places. In light of our findings on higher ETS exposure in participants reporting exposure to ETS at home, there is a need for public health educational programs and campaigns for all ages about the adverse health effects of ETS, and a need to develop and disseminate effective interventions to promote smoke free homes [27].

Finally, we recommend periodic surveys using biomarkers of ETS exposure (cotinine) for evaluating progress, or lack thereof, of policies to reduce exposure to tobacco smoke in non-smokers. Measurement of cotinine in urine, saliva or blood samples in the general population has been used by several countries, including the US, England, and Canada, to monitor exposure to ETS, and has been recommended by the World Health Organization as an advanced method for monitoring exposure to tobacco smoke [28]. In the US, information on ETS exposure using serum cotinine and urinary biomarkers has been collected since 1999, in the National and Nutrition Examination Survey. We recommend urinary cotinine measurements, since urine collection is non-invasive and is more sensitive than serum and saliva for detecting low level exposure [29]. Urinary cotinine has been used previously for population level ETS monitoring in Canada, Korea, and in the ‘Demonstration of a study to Co-ordinate and Perform Human Biomonitoring on a European Scale’. Indeed, the Public Health Laboratories at the Ministry of Health has developed analytical methods for measuring urinary cotinine. This will enable the Ministry of Health to continue research and surveillance on population level exposure to ETS, including in vulnerable populations such as children.

## Conclusions

We examined urinary cotinine concentrations in 194 adult participants in the National Health and Nutrition (RAV MABAT) Survey in 2015–2016. We found that exposure to ETS is widespread in non-smoking adults in Israel and is higher in Arab study participants, in former smokers, and in participants reporting exposure to ETS at home. We found that there has been no decrease in ETS exposure in non-smoking adults in Israel since 2011. To address the problem of widespread exposure to ETS in non-smokers, we recommend several changes in policy and practice including: (1) expansion of the current restrictions on smoking in public places to outdoor public areas, (2) increased enforcement on the ban on smoking in work and public places, (3) public health educational programs and campaigns for all ages about the adverse health effects of ETS, (4) interventions to promote smoke free homes, and (5) periodic surveys using biomarkers of ETS exposure (cotinine) for evaluating progress, or lack thereof, of policies to reduce exposure to tobacco smoke in non-smokers.

## Abbreviations

ETS: Environmental tobacco smoke
HBM: Human biomonitoring
INHIS-3: Israeli National Health Interview survey
LOD: Limit of detection
LOQ: Limit of quantification
